# Hematocrit in the first 2 hours of life and short-term outcomes in very preterm infants: A secondary analysis from a prospective cohort study

**DOI:** 10.1371/journal.pone.0336601

**Published:** 2025-12-01

**Authors:** Lanxiang Pu, Yuhong Li, Yongchao Tan, Ming Zhao, Meng Xue, Ning Jiang, Lei Huang, Zhaowen Chen

**Affiliations:** 1 Department of Pharmacy, Shandong Provincial Maternal and Child Health Care Hospital Affiliated to Qingdao University, Jinan, China; 2 Neonatal Intensive Care Units, Shandong Provincial Maternal and Child Health Care Hospital Affiliated to Qingdao University, Jinan, China; 3 Pediatrics department, Shandong Provincial Maternal and Child Health Care Hospital Affiliated to Qingdao University, Jinan, China; 4 Department of Gynaecology and Obstetrics, Shandong Provincial Maternal and Child Health Care Hospital Affiliated to Qingdao University, Jinan, China; Kobe University Graduate School of Medicine School of Medicine, JAPAN

## Abstract

**Background:**

Preterm birth, particularly very preterm birth (before 32 weeks of gestation), is a leading cause of neonatal morbidity and mortality. The early neonatal period is critical for preterm infants, with hematocrit levels serving as a important physiological indicator. We aimed to assess the relationship hematocrit in the first 2 hours of life and Short Outcomes in very preterm infants.

**Methods:**

The research was a prospective cohort study completed by Dongli Song et al. We acquired data from the DATA DRYAD website and utilized exclusively for secondary analysis. From January 2008 to April 2014, data were gathered prospectively from eligible infants at the Santa Clara Valley Medical Center. The main outcomes included any Intraventricular Hemorrhage (IVH), any Retinopathy of Prematurity (ROP), Necrotizing Enterocolitis (NEC), chronic lung disease (CLD), and late-onset sepsis (LOS). Secondary outcomes were any intubation and any transfusion. We used multivariable logistic regression analyses to calculate adjusted odd ratio (OR) with 95% CI.

**Results:**

This study included 312 patients in total. Hematocrit in the first 2 hours of life, considered as a continuous variable, was significantly associated with short-term outcomes in univariate analyses (P < 0.05). After adjusting for GA, BW, and sex, only any ROP, any intubation, and any transfusion were statistically significant. With adjustments for multiple factors, the odds ratios for any ROP and any transfusion in infants whose Hematocrit was 45 or more in the first two hours of life, compared to those with an HCT less than 45 were 0.43 (95% CI, 0.19 ~ 0.97, p = 0.043) and 0.29 (95% CI, 0.12 ~ 0.7, p = 0.006).

**Conclusions:**

Our study shows that higher HCT in the first 2 hours of life was statistically significant association with decreased ROP and blood transfusion in very preterm infants. Further clinical trials are necessary to confirm and validate this association.

## Introduction

The smaller the gestational age of a preterm infant, the higher the incidence and severity of neonatal complications [1–3]. The short-term complications most commonly seen are hypothermia, respiratory abnormalities, cardiovascular abnormalities, intracranial hemorrhage, hypoglycemia, necrotizing enterocolitis, infection, and retinopathy of prematurity. The presence of one or more of these complications increases the risk of death, and in survivors, increases the likelihood of long-term complications. Among these, retinopathy of prematurity is a significant cause of blindness that can be prevented in preterm infants [4].

The short-term outcomes of preterm infants are affected by factors such as gestational age, birth weight, sex, prenatal conditions, and the infant’s health status at birth and in the initial hours of life [3]. Interventions during the antenatal period, delivery process, and the immediate post-birth care also affected the short-term outcomes of preterm infants. More than twenty randomized trials have confirmed that a course of antenatal corticosteroid therapy administered to patients at risk for preterm birth decreases the incidence and severity of respiratory distress syndrome and mortality in their offspring [5]. Furthermore, antenatal corticosteroid treatment lowers the likelihood of developing necrotizing enterocolitis, intraventricular hemorrhage, and retinopathy of prematurity [6]. Hypothermia is associated with increased mortality and, among survivors, intraventricular hemorrhage, and pulmonary insufficiency and hemorrhage [7–13]. Other factors, multiple physiological indicators (including blood pressure, oxygenation, and urine output) may influence short-term outcomes of preterm infants [14].

Hematocrit (HCT) plays a crucial role in reflecting the proportion of red blood cells in blood and, therefore, directly influences oxygen-carrying capacity and microcirculation. In premature infants, immature physiology often leads to hemodynamic abnormalities, and changes in HCT levels are closely linked to various short-term adverse prognostic events.

Michael Fogarty et al. found that delayed cord clamping increased peak hematocrit and reduced the proportion of infants requiring blood transfusion by 10% [15]. Delayed cord clamping probably decreases the risk of mortality in preterm infants and slightly reduces the number of babies with any grade IVH [16]. While the benefits of interventions such as delayed cord clamping (DCC) on Hct are evident soon after delivery, the impact becomes more pronounced within minutes to hours following birth.

However, there is a notable scarcity of evidence specifically investigating the association between hematocrit in the first 2 hours of life and short-term outcomes in preterm infants. Utilizing previously published data, we performed a secondary data analysis for this study [17]. The objective is to explore the the association between hematocrit in the first 2 hours of life and short-term outcomes in preterm infants, including Intraventricular Hemorrhage (IVH), Retinopathy of Prematurity (ROP), Necrotizing Enterocolitis (NEC), chronic lung disease (CLD), late-onset sepsis(LOS), intubation, and transfusion.

## Methods

### Data source

The data for this study was obtained from the DATADRYAD platform (www.Datadryad.org), which offers open access to raw data downloads. Following Dryad’s Terms of Service, the Dryad data package (Dongli Song et al., 2016, Data from Duration of Cord Clamping and Neonatal Outcomes in Very Preterm Infants, Dryad, Dataset, https://doi.org/10.5061/dryad.4q3d3) must be cited when utilizing these data. The database file included the following variables for subsequent analysis: birth weight(BW), gestational age(GA), duration of cord clamping(duration of time for the neonate at birth before clamping the umbilical cord, DCC group), sex, multiple pregnancy, antenatal steroids >48hours prior to delivery, delivery mode, apgars at 1 and 5 minutes, delivery room intubation and positive pressure ventilation, delivery room chest compressions and medications, admission temperature in Celsius, hypothermia(<36 degree Celsius on admission), hematocrit in the first 2 hours of life, surfactant use, pneumothorax, any intubation (intubation any time during the NICU stay), any transfusion(any red blood cell transfusion in the NICU), peak bilirubin (mg/dl), polycythemia(Hematocrit >65%), death, any IVH(any grade 1–4 intraventricular hemorrhage), late onset sepsis(LOS), any ROP(any stage of retinopathy of prematurity, from 1 to 5), necrotizing enterocolitis(NEC), chronic lung disease(CLD), survival without major morbidities(severe intraventricular hemorrhage, severe retinopathy of prematurity, late onset sepsis, necrotizing enterocolitis or chronic lung disease).

### Study population

The original study reported by Song et al. was designed as a prospective cohort study to compared the effect of different duration of cord clamping and neonatal outcomes in very preterm Infants [17]. The Santa Clara Valley Medical Center(SCVM institutional review board approved this study as a quality improvement project, with a waiver for informed consent. The study involved 353 infants born extremely preterm, with gestational ages of less than 32 weeks. Infants with contraindications for DCC were excluded, including obstetric (placental or cord causes, i.e., placental abruption, placenta previa, cord avulsion and true knot) and fetal or neonatal causes (i.e., severely compromised infant without spontaneous respiration requiring immediate resuscitation after birth). NICU criteria for delivery room resuscitation, intubation, surfactant therapy and blood transfusion as well as the range of targeted oxygen saturation in preterm infants were not changed during the study period. The covariates considered in this study comprised demographic data (i.e., GA, BW, gender, 5 minute apgar, delivery mode and other potential variables).

In the current study, we explore the relationship between hematocrit in the first 2 hours of life and main outcomes of any ROP, any IVH, LOS, NEC, CLD. Additionally, we analyzed secondary outcomes, including any intubation, any transfusion. Participants missing data, such as hematocrit measurements within the first 2 hours of life or information on retinopathy of prematurity, were excluded from the study. Finally, 312 very preterm infants born at < 32 weeks gestation age were included in the secondary analysis.

### Statistical methods

Continuous variables were described using means and standard deviations for normally distributed data, and medians with interquartile ranges for skewed distributions. Categorical variables were summarized using frequencies and percentages. We employed the chi-square test for the comparison of categorical variables, the one-way ANOVA for normally distributed continuous variables, and the Kruskal-Wallis test for non-normally distributed continuous variables, respectively.

To assess the associations between hematocrit(HCT) in the first 2 hours of life and short outcomes in very preterm infants, multiple logistic regression models were employed. Both unadjusted and multivariate-adjusted models were utilized in the analysis.

All analyses were conducted using the statistical software package R.version 4.4.1 (http://www.R-project.org, The R Foundation) and Free Statistics analysis platform. A two-tailed statistical test was conducted, with a significance threshold set at p < 0.05.

## Results

### Baseline characteristics

**Fig 1:**
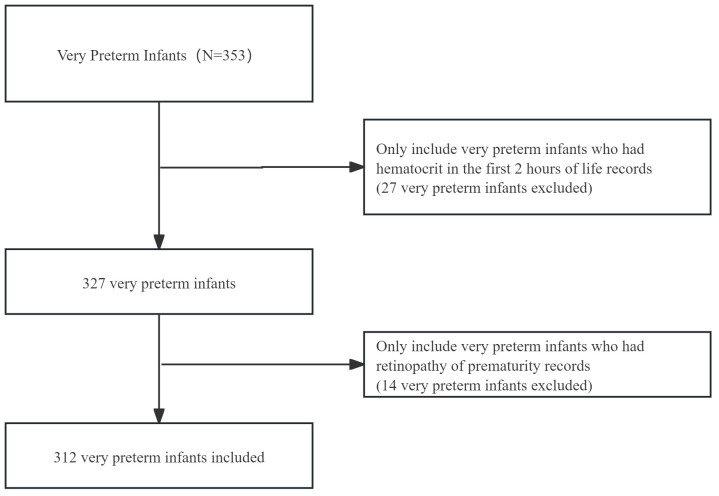
The flow chart of the study.

In total, 312 patients were enrolled in this study (Fig 1). Their average gestational age was 29.7 ± 2.5 weeks, and 195 (62.5%) were male. The average hematocrit in the first 2 hours of life was 48.4 ± 7.0. The incidence of retinopathy of prematurity (stage 1–3) was 25%. Table 1 presents the baseline characteristics of the infants divided into three groups based on the HCT in the first 2 hours of life(Q1 < 35%, Q2:35% ~ 55%, Q3: ≥ 55%) [18]. Among the patients in the Q1 group, the incidence of any intubation, LOS, any ROP and NEC were higher. Compared with the Q1 group, the Q2 group had higher any IVH and CLD. A multiple pregnancies constituted 19.6% of the study participants, while vaginal deliveries represented 40.1%. With a higher HCT in the first 2 hours of life, there was a gradual increase in the incidence of Survival without major morbidity (42.9%vs 74.8%vs 92.1%, P < 0.001).

**Table 1:** Baseline characteristics of participants.

Variables	Total (n = 312)	Q1(n = 7)	Q2 (n = 242)	Q3 (n = 63)	*p*
Birth Weight(g), Mean ± SD	1398.2 ± 451.8	1108.6 ± 389.2	1350.0 ± 461.6	1615.5 ± 338.0	< 0.001
Gestational age(week), Mean ± SD	29.7 ± 2.5	27.3 ± 2.7	29.4 ± 2.5	31.2 ± 1.7	< 0.001
Sex, n (%)					0.242
female	117 (37.5)	4 (57.1)	94 (38.8)	19 (30.2)	
male	195 (62.5)	3 (42.9)	148 (61.2)	44 (69.8)	
Duration of cord clamping, n (%)					0.104
30-45s	169 (54.2)	6 (85.7)	134 (55.4)	29 (46)	
60-75s	143 (45.8)	1 (14.3)	108 (44.6)	34 (54)	
Multiple pregnancy, n (%)	61 (19.6)	2 (28.6)	47 (19.4)	12 (19)	0.812
Vaginal delivery, n (%)	125 (40.1)	2 (28.6)	101 (41.7)	22 (34.9)	0.564
**Delivery room measures**					
apgar1, Mean ± SD	6.1 ± 2.2	3.4 ± 3.2	6.0 ± 2.2	6.7 ± 1.8	< 0.001
apgar5, Mean ± SD	7.6 ± 1.6	6.7 ± 1.6	7.5 ± 1.7	8.2 ± 1.1	0.006
Delivery room intubation and positive pressure ventilation	45 (14.4)	1 (14.3)	41 (16.9)	3 (4.8)	0.025
Delivery room Chest Compression and Medications					0.09
No	302 (96.8)	6 (85.7)	233 (96.3)	63 (100)	
Yes	10 (3.2)	1 (14.3)	9 (3.7)	0 (0)	
Admission temperature (°C), Mean ± SD	36.9 ± 0.5	37.1 ± 0.5	36.9 ± 0.5	37.0 ± 0.5	0.64
Admission Temperature <36˚C, n (%)	11 (3.5)	0 (0)	10 (4.1)	1 (1.6)	0.574
**Respiratory measures**					
Surfactant, n (%)	58 (18.6)	3 (42.9)	51 (21.1)	4 (6.3)	0.005
Intubation <24hr, %	78 (25.0)	2 (28.6)	69 (28.5)	7 (11.1)	0.007
Any Intubation	97 (31.1)	5 (71.4)	83 (34.3)	9 (14.3)	< 0.001
Antenatal Steroids > 48h, n (%)	217 (69.6)	4 (57.1)	167 (69)	46 (73)	0.566
**Hematological measures**					
Peak Bilirubin (mg/dL), Mean ± SD	8.5 ± 2.6	6.6 ± 4.2	8.3 ± 2.5	9.7 ± 2.2	< 0.001
Hematocrit >65%, n (%)					< 0.001
No	299 (95.8)	7 (100)	242 (100)	50 (79.4)	
Yes	13 (4.2)	0 (0)	0 (0)	13 (20.6)	
Any Red Blood Cell Transfusion, n (%)	82 (26.3)	5 (71.4)	75 (31)	2 (3.2)	< 0.001
< hematocrit in the first 2 hours of life,Mean ± SD	48.4 ± 7.0	32.4 ± 1.9	46.3 ± 4.8	58.3 ± 2.9	< 0.001
**Neonatal mortality and morbidities**					
Death, n (%)					1
No	311 (99.7)	7 (100)	241 (99.6)	63 (100)	
Yes	1 (0.3)	0 (0)	1 (0.4)	0 (0)	
Any IVH, n (%)	57 (18.3)	0 (0)	51 (21.1)	6 (9.5)	0.059
Severe IVH, n (%)					0.186
No	300 (96.2)	7 (100)	230 (95)	63 (100)	
Yes	12 (3.8)	0 (0)	12 (5)	0 (0)	
Late Onset Sepsis, n (%)	20 (6.4)	2 (28.6)	18 (7.4)	0 (0)	0.004
Any NEC, n (%)	11 (3.5)	1 (14.3)	9 (3.7)	1 (1.6)	0.209
CLD, n (%)	43 (13.8)	1 (14.3)	39 (16.2)	3 (4.8)	0.04
Any ROP, n (%)	78 (25.0)	6 (85.7)	70 (28.9)	2 (3.2)	< 0.001
Severe ROP, n (%)	15 (4.8)	1 (14.3)	14 (5.8)	0 (0)	0.051
Survival without major morbidity, n (%)	242 (77.6)	3 (42.9)	181 (74.8)	58 (92.1)	< 0.001

HCT(hematocrit in the first 2 hours of life): Q1 < 35%, Q2:35% ~ 55%, Q3: ≥ 55%

### Association between hematocrit in the first 2 hours of life and short-term outcomes

When the HCT in the first 2 hours of life was considered as a continuous variable, logistic regression analysis showed a statistically significant association between the HCT in the first 2 hours of life and the outcomes of any ROP, any IVH, LOS, NEC, CLD,any intubation and any transfusion in unadjusted models (Table 2). In the other models, this association between <2h HCT and any ROP remained statistically significant(OR=0.91, 95% CI, 0.86 ~ 0.97, P = 0.004; OR=0.92, 95% CI, 0.86 ~ 0.98, P = 0.014). Similarly, any transfusions remained statistically significant. Preterm infants with HCT in the first 2 hours of life < 45 compared to those with ≥45 were at a significant risk of any ROP, any IVH, LOS and CLD in unadjusted models. After adjusted birth weight, gestational age, sex, HCT in the first 2 hours of life was significantly associated with the outcomes of any ROP, any intubation and any transfusion. In the fully adjusted model (model III), We adjusted for covariates extracted from the raw data, including BW, GA, gender, multiple pregnancy, DCC group, antenatal steroids >48hours prior to delivery, vaginal delivery, 5 minute apgar, delivery room intubation and positive pressure ventilation, delivery room chest compressions and/or resuscitation medications, admission temperature in Celsius, surfactant, pneumothorax, peak bilirubin. We found that the relationship between <2h HCT and outcomes of any ROP and any transfusion still exists (OR=0.43, 95% CI 0.19 ~ 0.97, p = 0.043; OR=0.29, 95% CI 0.12 ~ 0.7, p = 0.006). However, in the model III, the association between <2h HCT and any intubation was not significant.

**Table 2:** Relationship between <2h Hct and Short-Term outcomes.

Short term outcomes	Model1		Model2		Model3	
**OR(95%cl)**	***p*-value**	**OR(95%cl)**	***p*-value**	**OR(95%cl)**	***p*-value**
**Primary outcomes**						
**Any Rop**						
Continuous variable	0.83 (0.79 ~ 0.88)	<0.001	0.91 (0.86 ~ 0.97)	0.004	0.92 (0.86 ~ 0.98)	0.014
< 45	Ref		Ref		Ref	
≥ 45	0.15 (0.09 ~ 0.27)	<0.001	0.38 (0.18 ~ 0.81)	0.012	0.43 (0.19 ~ 0.97)	0.043
**Any IVH**						
Continuous variable	0.93 (0.89 ~ 0.97)	0.002	1 (0.95 ~ 1.06)	0.918	1.02 (0.96 ~ 1.09)	0.458
< 45	Ref		Ref		Ref	
≥ 45	0.32 (0.18 ~ 0.58)	0.001	0.62 (0.31 ~ 1.24)	0.173	0.71 (0.32 ~ 1.58)	0.405
**LOS**						
Continuous variable	0.86 (0.8 ~ 0.93)	<0.001	0.95 (0.86 ~ 1.04)	0.246	0.96 (0.87 ~ 1.07)	0.494
< 45	Ref		Ref		Ref	
≥ 45	0.27 (0.11 ~ 0.69)	0.006	0.8 (0.28 ~ 2.34)	0.688	1.19 (0.34 ~ 4.17)	0.783
**NEC**						
Continuous variable	0.91 (0.83 ~ 0.99)	0.035	0.98 (0.88 ~ 1.09)	0.654	0.96 (0.85 ~ 1.07)	0.448
< 45	Ref		Ref		Ref	
≥ 45	0.53 (0.16 ~ 1.77)	0.302	1.21 (0.32 ~ 4.63)	0.777	1 (0.2 ~ 4.95)	1
**CLD**						
Continuous variable	0.87 (0.83 ~ 0.92)	<0.001	0.98 (0.9 ~ 1.06)	0.612	0.99 (0.89 ~ 1.09)	0.825
< 45	Ref		Ref		Ref	
≥ 45	0.23 (0.12 ~ 0.45)	<0.001	0.6 (0.22 ~ 1.6)	0.305	0.67 (0.2 ~ 2.2)	0.51
**Secondary outcomes**						
**Any intubation**						
Continuous variable	0.86 (0.82 ~ 0.9)	<0.001	0.93 (0.88 ~ 0.98)	0.006	0.93 (0.85 ~ 1.02)	0.114
< 45	Ref		Ref		Ref	
≥ 45	0.17 (0.1 ~ 0.29)	<0.001	0.35 (0.18 ~ 0.68)	0.002	0.54 (0.14 ~ 2)	0.356
**Any transfusion**						
Continuous variable	0.81 (0.77 ~ 0.86)	<0.001	0.89 (0.83 ~ 0.94)	<0.001	0.88 (0.82 ~ 0.95)	0.001
< 45	Ref		Ref		Ref	
≥ 45	0.14 (0.08 ~ 0.24)	<0.001	0.3 (0.14 ~ 0.65)	0.002	0.29 (0.12 ~ 0.7)	0.006

Model 1 was unadjusted model

Model 2 was adjusted for birth weight, gestational age, sex

Model 3 was adjusted for the variables in model 2 and further adjusted for multiple pregnancy, antenatal steroids >48hours prior to delivery, vaginal delivery, 5 minute apgar, delivery room intubation and positive pressure ventilation, delivery room chest compressions and medications, admission temperature in Celsius, surfactant, pneumothorax, DCC group, peak bilirubin.

## Discussion

In the current study, we found that lower HCT in the first 2 hours of life was statistically significant association with primary outcome of ROP in very preterm infants.

The findings of the present study are consistent with numerous other studies that have identified anemia as a risk factor for retinopathy of prematurity (ROP) [17,19,20]. Judith A. Englert et al. found that anemia does not independently influence the severity of retinopathy of prematurity (ROP) as a risk factor. Infants who experienced prolonged severe anemia, characterized by hemoglobin levels of 8 g/dL or hematocrit levels of 25%, exhibited milder forms of retinopathy of prematurity (ROP) compared to infants with less severe anemia [21]. Jayanta Banerjee et al. discovered a significant association between hemoglobin (Hb) levels and retinopathy of prematurity (ROP). However, after controlling for gestational age (GA) and birth weight (BW), this association was no longer statistically significant [22]. Furthermore, different to our finding, Brooks et al. reported no significant correlation between anemia or blood transfusion and the incidence or severity of retinopathy of ROP [23].

Anemia diminishes the blood’s capacity to transport oxygen [24]. Oxidative stress has the potential to activate VEGF receptor 2 (VEGFR2) signaling [25]. Reduced HCT levels contribute to tissue hypoxia and increased vascular endothelial growth factor (VEGF) release in the developing retinas of preterm infants. This relationship may elucidate the pathological significance of low HCT in the development of ROP.

Lower hematocrit in the first 2 hours of life was associated with the risk of NEC, IVH, LOS, and CLD, although this association was not significant when adjusted for GA, BW, et al. variables in the present study. Singh et al [26]. analyzed records of 111 preterm infants with NEC and 222 controls, finding that lower hematocrit was linked to NEC (OR 1.10, p = 0.01) in multivariate models. Anemia may hinder the development of vascular autoregulation in the premature intestine, increasing the risk of ischemic injury and potentially leading to NEC [27,28]. Belma Saygili Karagol et al. found that a higher initial hematocrit (Hct) at birth is associated with a decreased incidence of periventricular/intraventricular hemorrhage (P/IVH) in extremely low birth weight (ELBW) infants [29]. An initial Hct < 45% doubled the risk of P/IVH [30]. Michael Fogarty et al.‘s meta-analysis supports our view that while delayed cord clamping can raise newborns’ hematocrit and decrease blood transfusion needs, it does not reduce the incidence of LOS and CLD [31].

This study found that preterm infants with higher hematocrit (HCT) levels had significantly reduced transfusion requirements. This is consistent with existing transfusion guidelines that promote restrictive transfusion approaches, focusing on maintaining suitable HCT levels to enhance oxygen delivery and minimize unnecessary transfusions, thus decreasing transfusion-related risks [32–34].

Our study also has several limitations. Firstly, it was conducted as a single-center observational study, despite the prospective collection of data. Secondly, given that our study was a secondary analysis, we are unable to ensure comprehensive data quality monitoring and control over variables. Thirdly, although 41 infants were excluded due to missing data, this represents a minor fraction of the study population and is unlikely to significantly affect the study’s findings. Lastly, our analysis was limited to examining the relationship between hematocrit levels within the first two hours of life and the incidence of short-term outcomes, without further exploration of its impact on phases of disease.

## Conclusion

In conclusion, our study demonstrates a statistically significant association between higher hematocrit levels in the first two hours of life and the primary outcome of reduced incidence of retinopathy of prematurity (ROP), as well as the secondary outcome of decreased blood transfusion requirements in very preterm infants. This finding holds considerable importance for clinicians in the management of very preterm infants, as it may contribute to reducing the incidence of ROP. However, further clinical trials are necessary to confirm and validate this association.
